# Ablation of Atrial Fibrillation in Patients with Hypertension—An Analysis from the German Ablation Registry

**DOI:** 10.3390/jcm9082402

**Published:** 2020-07-27

**Authors:** Maura M. Zylla, Matthias Hochadel, Dietrich Andresen, Johannes Brachmann, Lars Eckardt, Ellen Hoffmann, Karl-Heinz Kuck, Thorsten Lewalter, Burghard Schumacher, Stefan G. Spitzer, Stephan Willems, Jochen Senges, Hugo A. Katus, Dierk Thomas

**Affiliations:** 1 Department of Cardiology, Medical University Hospital, Im Neuenheimer Feld 410, 69120 Heidelberg, Germany; Maura.zylla@med.uni-heidelberg.de (M.M.Z.); sekretariat.katus@med.uni-heidelberg.de (H.A.K.); 2 HCR (Heidelberg Center for Heart Rhythm Disorders), Medical University Hospital Heidelberg, Im Neuenheimer Feld 410, 69120 Heidelberg, Germany; 3 DZHK (German Center for Cardiovascular Research), partner site Heidelberg/Mannheim, Medical University Hospital Heidelberg, 69120 Heidelberg, Germany; 4 Stiftung Institut für Herzinfarktforschung, IHF, Bremserstraße 79, 67063 Ludwigshafen, Germany; Hochadel@ihf.de (M.H.); senges@stiftung-ihf.de (J.S.); 5 Department of Cardiology, Vivantes Hospital, Klinikum am Urban, Dieffenbachstraße 1, 10967 Berlin, Germany; Dietrich.Andresen@jsd.de; 6 Department of Cardiology, Hospital Coburg, Ketschendorfer Str. 33, 96450 Coburg, Germany; jobraco@gmx.de; 7 Division of Electrophysiology, Department of Cardiovascular Medicine, University Hospital Münster, Albert-Schweitzer-Campus 1, 48149 Münster, Germany; lars.eckardt@ukmuenster.de; 8 Department of Cardiology/Intensive Care Medicine, Heart Center Munich-Bogenhausen, Englschalkinger Str. 77, 81925 Munich, Germany; Ellen.Hoffmann@muenchen-klinik.de; 9 Department of Cardiology, Asklepios Hospital St. Georg, Lohmühlenstraße 5, 20099 Hamburg, Germany; Karl-Heinz.Kuck@uksh.de; 10 Klinik für Kardiologie und Internist, Intensivmedizin, Peter Osypka Herzzentrum, Internistisches Klinikum München Süd, Am Isarkanal 36, 81379 Munich, Germany; thorsten.lewalter@ikms.de; 11 Department of Cardiology, Herz- und Gefäßklinik, Salzburger Leite 1, 97616 Bad Neustadt/Saale, Germany; bschumacher@westpfalz-klinikum.de; 12 Praxisklinik Herz und Gefäße, Forststraße 3, 01099 Dresden, Germany; prof.spitzer@praxisklinik-dresden.de; 13 Institute of Medical Technology, Brandenburg University of Technology Cottbus-Senftenberg, Universitätsplatz 1, 01968 Senftenberg, Germany; 14 Department of Cardiology/Electrophysiology, University Heart Center, Martinistraße 52, 20251 Hamburg, Germany; s.willems@asklepios.com

**Keywords:** catheter ablation, atrial fibrillation, arterial hypertension, complications, registry, long-term follow-up

## Abstract

Background: Hypertension (HTN) constitutes a risk factor for the development of atrial fibrillation (AF), as well as for thromboembolic and bleeding events. We analysed the outcome after catheter ablation of AF in HTN in a cohort from the prospective multicenter German Ablation Registry. Methods: Between 03/2008 and 01/2010, 626 patients undergoing AF-ablation were analysed. Patients diagnosed with HTN (*n* = 386) were compared with patients without HTN (*n* = 240) with respect to baseline, procedural and long-term outcome parameters. Results: Patients with HTN were older and more often presented with persistent forms of AF and cardiac comorbidities. Major and moderate in-hospital complications were low. At long-term follow-up, major cardiovascular events were rare in both groups. Rates of AF-recurrence, freedom from antiarrhythmic medication and repeat ablation were not statistically different between groups. Most patients reported improvement of symptoms and satisfaction with the treatment. However, patients with HTN more frequently complained of dyspnea of New York Heart Association (NYHA) class ≥ II and angina. They were more often rehospitalized, particularly when persistent AF had been diagnosed. Conclusion: Catheter ablation of AF is associated with low complication rates and favorable arrhythmia-related results in patients with HTN. Residual clinical symptoms may be due to cardiac comorbidities and require additional attention in this important subgroup of AF-patients.

## 1. Introduction

Arterial hypertension (HTN) constitutes a major risk factor for the development of atrial fibrillation (AF) and incidence of AF is significantly increased in patients with HTN [1]. Due to its high prevalence in the general population, hypertension has been shown to be the most important contributor to AF-development [2]. HTN and AF also share several risk factors, such as male sex, obesity and sleep apnea contributing to the widespread coexistence of these two conditions. In study-cohorts of AF-patients, the prevalence of HTN accounted for ~60 to over 90%. [1,3,4,5,6,7]. Experimental studies have proposed several pathomechanisms by which HTN increases susceptibility for AF, including atrial enlargement and hypertrophy due to left ventricular diastolic dysfunction, fibrotic remodeling and inflammation leading to enhanced heterogeneity of atrial conduction, alterations in calcium handling and variable changes in effective refractory periods [8,9,10,11,12,13,14]. In patients with known AF, HTN also promotes progression from paroxysmal to persistent AF [15].

Additionally, HTN increases the risk for thromboembolic events in AF-patients. As an independent risk factor, HTN increases the risk of stroke and its recurrence and significantly influences prognostic outcome in AF [16,17]. Furthermore, bleeding complications are increased in patients with HTN and AF, in particular intracranial hemorrhage [18]. Subsequent analyses from the “Apixaban versus Warfarin in Patients with Atrial Fibrillation”-trial (ARISTOTLE), the “Rivaroxaban versus Warfarin in Nonvalvular Atrial Fibrillation”-trial (ROCKET) and the “Dabigatran versus Warfarin in Patients with Atrial Fibrillation”-trial (RE-LY) have demonstrated elevated rates of ischemic stroke, major bleeding complications and hemorrhagic stroke in AF-patients with HTN [4,19,20]. Therefore, HTN contributes to both the CHA_2_S_2_D-Vasc-Score and the HAS-BLED-Score. In summary, HTN affects development, prognosis and therapy in patients with AF.

Catheter ablation has emerged as a common interventional therapy for symptomatic AF. We analyzed a patient cohort from the prospective multicenter German Ablation Registry undergoing catheter ablation of AF in order to investigate the long-term effects of HTN on AF-recurrence, freedom from antiarrhythmic medication, repeat ablation, major cardiovascular events, hospitalizations, symptoms and patient satisfaction.

## 2. Materials and Methods

### 2.1. The German Ablation Registry

The German Ablation Registry was conducted as a prospective, multicenter registry study, driven by scientific interest of the participating centers. A total of 55 German centers participated in contributing data to the registry. The study was performed in accordance with the Declaration of Helsinki. The registry has been approved by the ethics committee of the medical association of Rheinland-Pfalz under the study ID 837.026.07 (5561) (approval date: 7 March 2007). Project management and clinical monitoring were carried out by the Stiftung Institut für Herzinfarktforschung (IHF, Ludwigshafen, Germany) which was the central contract research organization for the study.

### 2.2. Data Collection and Patient Selection

Patient-specific and procedural data were collected after obtained written informed consent. Documentation and data acquisition were voluntary and were carried out on an electronic internet-based case report form system. Transmitted data was confidential and encrypted with a secure socket layer. Centers were asked to enrol consecutive patients undergoing ablation during the participation period.

Demographic and clinical baseline parameters, procedural data and complications were collected in the registry. For this study, a total of 626 patients undergoing catheter ablation of AF between 03/2008 and 01/2010 and with complete long-term follow-up data were included in the analysis. Patients diagnosed with HTN (*n* = 386) were compared with patients without HTN (*n* = 240) with respect to baseline, procedural and long-term clinical outcome parameters. The presence of arterial hypertension was diagnosed according to the participating centers’ medical records.

Study participants with primary electrical heart disease and undergoing repeat AF ablation procedures were excluded. The choice of ablation methods and techniques, as well as postablation therapy was left to the discretion of the treating physician and carried out according to the participating centers’ standards. Preclassified ablation approaches were circumferential or segmental pulmonary vein (PV) ablation, linear lesions and ablation of complex fractionated atrial electrograms. All procedure-related adverse events requiring therapeutic intervention or hospitalization were recorded as complications. Complications were classified as major, moderate and minor. Major nonfatal complications included stroke, myocardial infarction and major bleeding. Moderate complications comprised transient ischemic attack (TIA), resuscitation, peripheral vascular complications, third degree AV block, phrenic nerve palsy, pneumothorax, hemothorax, pericardial effusion, pulmonary embolism, PV stenosis, emergency cardiac surgery and atrio-esophageal fistula. Minor complications were defined as bleeding without need for intervention, new first- or second-degree AV block or bundle branch block.

After ≥1 year from enrolment, a telephone follow-up was carried out. Patients were asked about symptoms, complications, hospitalizations and medical or interventional therapy during follow-up as well as their satisfaction with the ablation treatment since the index procedure. Patient satisfaction was categorized as treatment perceived as “successful”, “partially successful” and “not successful”, and patients were asked to choose the category applicable. Rates of AF recurrence refer to arrhythmia-recurrence that was reported as electrocardiography (ECG)- documented by a physician.

### 2.3. Statistical Methods

Continuous data are reported as median (with interquartile range) or mean  ±  standard deviation (SD), categorical data are presented as percentages. The Mann–Whitney–Wilcoxon test was applied for between-group comparisons of ordinal or metrical variables, and the Pearson chi-square test for binary variables, or Fisher’s exact test in case of infrequent events. Rates of nonfatal follow-up events are reported as rates among survivors. Adjusted odds ratios with 95%-confidence intervals for follow-up outcomes were calculated for paroxysmal and (long-standing) persistent AF using multiple logistic regression models including HTN, type of AF and their interaction, as well as age, sex and other patient characteristics (Table 1) with distributions significantly differing between the patient groups. All statistical comparisons were two-sided. *p* values ≤ 0.05 were considered statistically significant. The statistical analysis was performed at the biometrics department of the IHF (Ludwigshafen) using SAS 9.4 software (SAS Institute, Cary, NC, USA).

## 3. Results

### 3.1. Baseline Characteristics

Of 626 patients included in this study, HTN had been diagnosed in 386 patients. The majority of patients in both groups were male, similar to previous AF cohorts [21,22,23]. Patients with HTN were older and more often suffered from other cardiac comorbidities, in particular coronary artery disease and previous myocardial infarction (Table 1). Valvular heart disease was present in 10.9% of patients with HTN and 7.5% without HTN (*p* = 0.16). Left ventricular ejection fraction (LVEF) was preserved in the majority of cases and there was no statistically significant difference regarding LVEF in both groups (Table 1). A small number of patients had been diagnosed with cardiomyopathies (Table 1), mainly dilated cardiomyopathy (HTN: 76.9% of patients with cardiomyopathies, no HTN 80%, *p* = 0.86). HTN was associated with higher rates of diabetes, kidney failure and peripheral artery disease (Table 1). Mean CHA_2_DS_2_-Vasc-Score was 2.4 ± 1.1 in patients with HTN (recorded in 86.5% of cases) and 0.9 ± 1.0 in patients without HTN (recorded in 91.2% of cases) (*p* < 0.001). In patients with HTN, persistent AF was more common whereas paroxysmal atrial fibrillation was more often present in patients without arterial hypertension (Table 1). The majority of patients presented with palpitations as the main symptom (HTN: 96.9%; no HTN: 96.3%, *p* = 0.66) and had AF-symptoms at least once a month (HTN: 91.7%; no HTN: 89.2%, *p* = 0.29).

### 3.2. Ablation Procedure and Procedure-Related Complications

The main target for AF ablation was pulmonary vein isolation (PVI) in all cases. The majority of procedures were performed using radiofrequency ablation (Table 2). Procedural success defined as successful isolation of all pulmonary veins was achieved in 97.4% (HTN) and 96.4% (no HTN), respectively (*p* = 0.59). Additional linear lesions were performed in 21.8% of patients with HTN and in 15.0% without HTN (*p* = 0.037). Ablation of complex fractionated potentials was carried out in 13.0% of cases with HTN and 3.8% of patients without hypertension (*p* < 0.001). Three-dimensional mapping was employed in 63.0% of patients with HTN and 51.7% of patients without HTN (*p* = 0.005), in the remaining cases conventional mapping was used. There was no statistically significant difference in procedure duration between the two groups (HTN: Median 180 min [P_25_: 153 min; P_75_: 200 min]; no HTN: 165 min [P_25_: 125 min; P_75_: 210 min], *p* = 0.094). Major complications were rare events and consisted of six cases of severe bleeding complications in the HTN-group (Table 2). Regarding moderate complications, few cases of vascular complications at the access site and of pericardial effusion were recorded in both groups. There was no statistically significant difference in overall moderate complication rates between the two groups. Minor complications were increased in patients with HTN, consisting of minor bleeding complications without the need for medical intervention. In the HTN-group, one patient (0.3%) underwent a pacemaker implantation due to procedure-related cause. In the patient group without HTN, two patients (0.8%) received a pacemaker, of which one was implanted due to procedure-related reasons (*p* = 0.56). Details regarding this case and whether this incident was due to iatrogenic AV-block were not provided by the registry. During the index hospital stay, consecutive secondary arrhythmias occurred in 1.0% of patients with HTN, consisting of four cases of atrial tachycardia, and in 1.3% of patients without HTN, consisting of one case of detected AVNRT, one case of atrial flutter and one case of atrial tachycardia (*p* = 0.21). Patients with hypertension were more often discharged on antiarrhythmic medication, in particular class III antiarrhythmic drugs (Table 2). Additionally, clopidogrel, statins, diuretics, angiotensin-converting-enzyme-(ACE)-inhibitors and angiotensin-II-receptor-blockers (ARBs) were more often used in this patient group (Table 2).

### 3.3. Long-Term Follow-Up

Median follow-up in the patient group with hypertension was 498 days (P_25_: 465 days; P_75_: 516 days) and 491 days in the subgroup without HTN (P_25_: 439 days; P_75_: 518 days; *p* = 0.15). Follow-up rates were high in both groups (HTN: 92.7%; no HTN: 93.8%; *p* = 0.63). Mortality rates at follow-up were low (HTN: 0.8%; no HTN: 0.9%). All deaths (HTN: *n* = 3; no HTN: *n* = 2) were attributed to cardiac causes. Major nonfatal cardiovascular events occurred in 1.1% of patients with HTN and 0.9% without HTN (Fisher’s exact test *p* = 1.00), consisting of one case of myocardial infarction and one major bleeding event in the HTN-group, and one stroke and one case of major bleeding complication in the patient group without HTN. Moderate complications were present in 10.1% of patients with HTN and 7.6% without HTN (*p* = 0.37), consisting mainly of complications at the puncture site (HTN: 5.2%; no HTN: 2.8%, *p* = 0.20), moderate bleeding complications (HTN: 2.6%; no HTN: 1.9%; *p* = 0.77) and the need for coronary revascularization by PCI (HNT: 2.0%; no HTN: 2.4%; *p* = 0.77).

**Figure 1:**
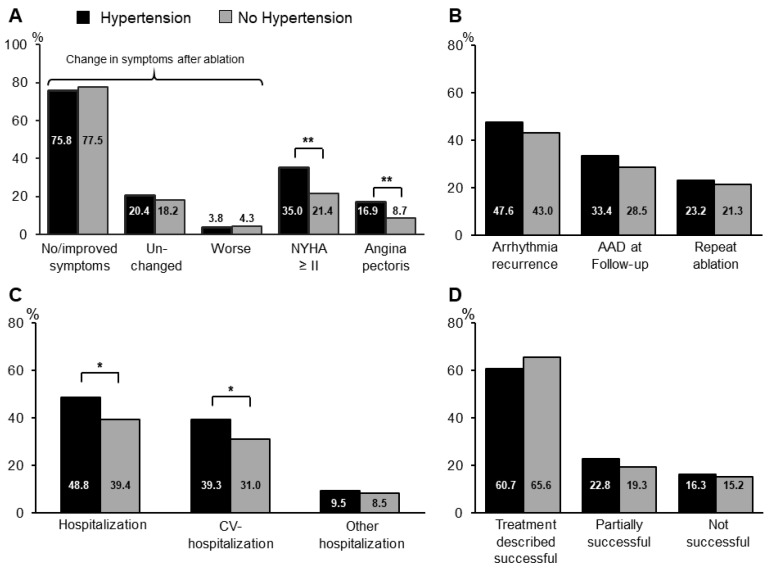
Clinical outcome at long-term follow-up. (**A**): Symptoms at long-term follow-up. Patients were asked to classify the change in symptom burden after atrial fibrillation (AF) ablation into the categories “No/improved”, “unchanged”, and “worse”. NYHA = New York Heart Association. (**B)**: Arrhythmia-related outcome at long-term follow-up. AAD = antiarrhythmic drug (**C**): Rehospitalization rates during long-term follow-up period. CV = cardiovascular. The bar graphs for “CV-hospitalizations” and “Other hospitalizations” reflect subgroups of the overall hospitalization-rates depicted in the first bar graph. (**D**): Patient satisfaction at follow-up (* *p* < 0.05; ** *p* < 0.01).

The majority of patients in both groups reported improvement of or freedom from symptoms (75.8% vs. 77.5%, *p* = 0.65) at long-term follow-up after AF ablation (Figure 1A). Rates of AF recurrence, freedom from AAD and repeat ablation were not statistically different between groups (Figure 1B). However, hospitalization rates were increased in the HTN-group at follow-up and occurred predominantly due to cardiovascular reasons (Figure 1C). Pacemaker-implantations were performed in 2.5% of patients with HTN and 1.8% of patients without HTN during the course of the long-term follow-up (*p* = 0.56). In spite of overall improvement of symptoms after AF ablation, more patients in the HTN-group reported shortness of breath NYHA ≥ II and angina (Figure 1A). Nevertheless, the majority of patients reported overall satisfaction with the treatment (Figure 1D). At follow-up, patients with HTN were more often treated with beta-blockers, diuretics, statins and ACE-inhibitors/ARBs (Table 3). Oral anticoagulation had been discontinued in more than half of all patients in both groups at long-term follow-up (Table 3).

Additional analyses were performed for patients with paroxysmal and persistent/long-standing persistent AF employing logistic regression for evaluation of type of AF and age as predictor of clinical outcomes. Adjusting for the different rates of paroxysmal AF and for age in the two groups, there was still no significant difference regarding arrhythmia recurrence (*p* = 0.39), freedom of AAD (*p* = 0.71), reablation rates (*p* = 0.69), no or improved symptoms (*p* = 0.97) and patient satisfaction (*p* = 0.56). However, differences regarding rehospitalization rates and symptoms were more pronounced in patients with persistent or long-standing persistent AF (Table 4). In logistic regression analyses after adjustment for differences in baseline parameters, HTN was a significant predictor for rehospitalization (*p* = 0.045) and presence of dyspnea (*p* = 0.042) or angina (*p* = 0.025) in the subgroup of patients with persistent/long-standing persistent AF.

## 4. Discussion

The present analysis of a large patient cohort from multiple centers shows no compromising effect of HTN on the arrhythmia-related long-term outcome after AF ablation in contrast to previous analyses. Adequate risk factor control and additional ablation targets with respect to a more complex arrhythmogenic substrate may have contributed to this beneficial effect in this cohort. However, rehospitalization rates and presence of cardiac symptoms were increased in patients with HTN during long-term follow-up. Particularly, patients with persistent forms of AF at baseline could be identified as a patient subgroup at risk for adverse events at long-term follow up. Therefore, this study offers important observations from a real-world cohort with a high long-term follow-up rate.

### 4.1. Preprocedural Differences and Periprocedural Outcome

Patients with HTN undergoing AF ablation were older than patients without HTN, however, age-distribution was still comparable to typical AF cohorts from previous studies focusing on AF-ablation [21,22]. HTN was associated with a higher prevalence of diabetes and renal insufficiency as additional cardiovascular risk factors. Consequently, rates of coronary artery disease and previous myocardial infarction, as well as peripheral artery disease, were elevated in patients with HTN. The association of these cardiac and noncardiac conditions with the risk factor of HTN characterizes real-world cohorts of patients with HTN.

In patients with HTN, persistent forms of AF were more common than in patients without diagnosed HTN. This observation is consistent with previous studies showing an association of HTN with progression of paroxysmal to persistent AF [15]. More extensive use of 3-D-mapping and ablation of additional targets may have been a sign of more complex electrophysiologic substrate expected or detected in the HTN-group. Nevertheless, procedure duration was comparable between the two groups. The notion of a higher arrhythmogenicity in patients with HTN may also have been promoted by coexistent cardiac conditions. In particular, coronary artery disease and ischemic heart disease may have additionally enhanced more extensive structural remodelling. Importantly, however, left ventricular function was not different between groups and the majority of patients displayed preserved systolic function. Similarly, valvular heart disease, which may promote structural changes both of the atria and the ventricles, was not more common in patients with HTN. Therefore, the presence of HTN itself may have influenced arrhythmogenicity of the atrial substrate as perceived by the operator leading to more elaborate ablation strategies. The association of hypertension and arrhythmogenic atrial remodelling requiring substrate modification has been shown in previous studies [24]. In line with this finding, patients with increased LA pressures, as often associated with HTN, also display more extensive arrhythmogenic substrate and higher rates of arrhythmia recurrence [25]. After successful AF ablation, patients with elevated LA pressure seem to show a greater benefit regarding quality of life than patients without LA pressure elevation [25]. However, whether the direct hemodynamic effects of HTN by elevated atrial strain and atrial pressure or indirect influences by HTN-promoted cardiac comorbidities play the leading role for atrial remodelling has to be evaluated in experimental studies aimed at mechanistic analyses.

In patients with HTN, more frequent use of diuretics and ACEIs/ARBs at discharge reflects therapeutic efforts in controlling this important cardiovascular risk factor. The increased use of statins and clopidogrel in patients with HTN is consistent with the increased prevalence of vascular disease.

The majority of patients received anticoagulation therapy with vitamin K antagonists at discharge from index ablation which were commonly used for thromboembolic prevention at the time of data collection. Since then, NOACs have emerged as a safe alternative the vitamin K antagonists and NOAC therapy has been proven to be easy to manage periprocedurally [26]. Hypertension has been shown to increase bleeding risk under oral anticoagulation, also when using NOACs, in patients with HTN [19,20]. Our study also shows increased periprocedural bleeding complications in patients with HTN; however, the majority were minor bleeding events without need for medical intervention. Additional use of antiplatelet therapy points to either perceived need for protection against atherosclerosis-triggered events or recent PCI in a small subgroup of patients with HTN. However, the underlying indication for antiplatelet therapy in individual cases cannot be extracted from these registry data. Combination therapy may also have promoted bleeding complications. With now widespread use of NOACs and new emerging therapy regimens after PCI with less extensive use of triple versus dual therapy after PCI, new, prospective trials are needed to evaluate the optimal therapy in patients with coronary artery disease undergoing AF ablation [26].

### 4.2. Long-Term Outcome after AF Ablation

Hypertension has previously been identified as a risk factor for arrhythmia recurrence after AF ablation [27,28] In particular, uncontrolled hypertension seems to promote arrhythmogenic substrate in patients undergoing AF ablation whereas adverse remodelling processes seem to be attenuated in well-controlled hypertension [24,29]. In contrast to these previous studies, HTN was not associated with a compromised arrhythmia-related outcome in our registry analysis. The majority of patients reported freedom from or improvement of AF-related symptoms at long-term follow-up and satisfaction with the outcome after ablation therapy. Rates of arrhythmia recurrence, use of AAD und reablation were not statistically different between patients with and without concomitant HTN, which stands in contrast to previous observations [27,28]. One possible explanation may be the high prevalence of antihypertensive medication, in particular ACE/ARBs, which may have controlled adverse influences of hypertension and limited structural remodelling in this cohort. Inhibition of the renin-angiotensin-aldosterone-system (RAAS) has been proposed to exert positive effects on atrial structural remodelling [30]. However, clinical studies have rendered mixed results as to the role of RAAS-inhibition for preventing arrhythmia recurrence [31,32,33,34,35,36]. With respect to antihypertensive therapy in AF-patients, the “Substrate Modification With Aggressive Blood Pressure Control”-study (SMAC-AF) showed no additional benefit from aggressive blood pressure control (<120/80 mmHg) regarding arrhythmia recurrence after AF ablation in comparison to standard blood pressure targets of (<140/90 mmHg) [37]. However, aggressive antihypertensive treatment showed a positive effect on rhythm outcome in older patients (age > 61 years). Therefore, also a subgroup of patients in the HTN-group from our registry study may be benefited from adequate or event intensified antihypertensive medication, highlighting the need for rigorous risk factor control in these patients. In general, European consensus statements regard AF as one of the manifestations of hypertensive heart disease and endorse adequate antihypertensive therapy in these patients [38].

The favorable rhythm outcome in the HTN-group may also have been supported by tailored ablation targets, including more extensive ablation of non-PVI-triggers in patients with HTN and of possibly more complex arrhythmogenic substrate. However, prospective randomized trials are needed to evaluate the best ablation strategy in individual AF-patient subgroups with different coexistent conditions.

Despite a mean CHA_2_DS_2_-Vasc-Score > 2 in patients with HTN, anticoagulation was discontinued in more than half of all patients reflecting insufficient thromboembolic prevention in this real-world cohort. In particular, in the light of a recurrence rate of >50% in this study, (reflecting only symptomatic forms of AF recurrence) this is a striking observation. It should motivate physicians to thoroughly evaluate the individual thromboembolic risk also after initially successful AF ablation and inform patients about the individual benefit-and-risk-ratio of oral anticoagulation in the light of a still relevant rate of arrhythmia recurrence. Prospective, randomized data regarding the safety of discontinuing oral anticoagulation after initially successful AF ablation in low risk patients are needed as most therapy decisions and regimens in this subgroup are still determined based on repeated Holter-ECGs, with possibly insufficient sensitivity and individual patient preference. This study may stress the importance of this issue for patients and physicians and may motivate respective study efforts.

In spite of comparable arrhythmia-associated outcome, rehospitalization rates were elevated in patients with HTN, predominantly for cardiovascular reasons. In line with this observation, patients in the HTN-group more often reported angina and shortness of breath, possibly leading to hospitalization for further diagnostic evaluation. These differences were particularly pronounced in patients with persistent AF. Cardiac comorbidities, especially the presence of coronary artery disease may have led to cardiovascular-related hospitalizations during follow-up. However, rates of revascularization during follow-up were not different between groups, as were rates of device-implantations. Systolic LV function was not different between groups at baseline and was preserved in the majority of cases. However, a deterioration of LV function, e.g., due to tachycardiomyopathy in patients with persistent AF or heart failure with preserved ejection fraction cannot be excluded as follow-up echocardiography data were not collected. Based on the registry data, a predominant reason for rehospitalization in patients with HTN cannot be identified. Nevertheless, these observations show that patients with HTN and particularly with persistent forms of AF are more often rehospitalized during the long-term period after AF ablation and more often suffer from cardiac symptoms. This effect was apparently nondependent on rhythm-associated outcome which was not significantly different between groups. Therefore, persistent AF in HTN may be a marker for compromised symptomatic outcome. Physicians should be aware of this subgroup of patients possibly needing closer follow-up, apart from AF-specific therapies, in order to early detect decline in clinical condition or cardiac function.

### 4.3. Limitations

This study carries inherent limitations of a registry analysis. Centers were asked to enrol consecutive patients on a voluntary basis. Ablation methods and periprocedural therapy were chosen according to the respective center’s standards and were not standardized by the study protocol. Additional risk factors associated with AF-development and -recurrence (e.g., overweight, sleep apnea) were not registered and could not be corrected for. Furthermore, additional echocardiographic data regarding right and left atrial dimensions would have improved baseline characterization of this cohort but were not included in the study protocol. Although most rehospitalization events during the long-term follow-up were due to cardiovascular reasons, the exact diagnosis leading to hospitalization cannot be extracted from the registry data. However, a higher rate of cardiac symptoms such as dyspnea or angina may have triggered the need for further evaluation. The diagnosis of HTN was based on the information from the respective participating center with respect to the patients’ comorbidities. Detailed information regarding the severity and cause of arterial hypertension as well as the efficiency of current medical therapy are missing, constituting a major limitation regarding a more distinguished insight into the interplay of AF and hypertension. A smaller subgroup of patients without reported HTN also received therapy with diuretics, ACE-inhibitors/ARBs and betablockers. The respective indications for this medication in this subgroup are not known, constituting a limitation of this study. Baseline differences between the two groups regarding age and comorbidities may have influenced outcome and clinical course. We cannot exclude that differences between groups affected the parameters presented in this study despite logistic regression analyses. However, as these factors are regularly associated with HTN in everyday clinical practice, we aim to depict a real-world patient cohort of this important subgroup of AF patients.

## 5. Conclusions

Patients with and without HTN show low complication rates and a similar outcome as to arrhythmia-related endpoints after AF-ablation in this analysis of a large multicenter cohort. Medical therapy of HTN and associated conditions as well as tailored ablation strategies may have contributed to the positive outcome. However, rehospitalizations and residual cardiac symptoms were particularly increased in patients with HTN and persistent AF and may require additional attention in this important subgroup of AF patients.

## Figures and Tables

**Table 1:** Baseline characteristics.

	HTN (*n* = 386) (%)	No HTN (*n* = 240) (%)	*p* Value
**Male**	64.0	67.9	0.31
**Age (years), mean ± std**	62.5 ± 8.8	56.5 ± 11.9	<0.001
**Paroxysmal AF**	64.5	72.5	0.038
**Persistent AF**	26.7	21.7	0.16
**Long-standing** **persistent AF**	8.8	5.8	0.17
**Cardiac comorbidities**	43.3	26.7	<0.001
Coronary artery disease	23.1	10.4	<0.001
Previous myocardial infarction	5.4	1.7	0.019
Cardiomyopathy	3.4	4.2	0.61
**Left ventricular function**			0.57
LVEF > 50%	87.0	88.6	
LVEF 41–50%	7.5	6.4	
LVEF 31–40%	3.8	3.7	
LVEF ≤ 30%	1.7	1.4	
**Pacemaker**	4.4	4.2	0.99
**Implanted Cardioverter Defibrillator (ICD)**	1.3	2.1	0.89
**Cardiac Resynchronization Therapy (CRT)-device**	0.5	0.0	0.44
**Diabetes**	13.2	2.5	<0.001
**Renal insufficiency**	3.7	0.8	0.029
**Peripheral artery disease**	2.1	0.0	0.024
**History of stroke**	5.5	5.0	0.77
**COPD**	1.3	2.1	0.47

AF = atrial fibrillation, CM = cardiomyopathy, COPD = chronic obstructive pulmonary disease, LVEF = left ventricular ejection fraction, std = standard deviation; Left ventricular function was recorded in recorded in *n* = 345 in the HTN-group and *n* = 219 in the patient group without HTN. Data are presented as percentages of patients unless otherwise stated.

**Table 2:** Procedure and index hospital stay.

	HTN (%)	No HTN (%)	*p* Value
**Radiofrequency ablation**	80.6	75.0	0.099
**Cryoballoon ablation**	17.1	24.2	0.031
**Other**	2.3	0.8	0.17
**Major complications**	1.6	0.0	0.087 *
Myocardial infarction/stroke	0.0	0.0	n.d.
Major bleeding	1.6	0.0	0.087 *
**Moderate complications**	4.5	3.0	0.40 *
Transient ischemic attack	0.3	0.0	1.00 *
Aneurysm/arteriovenous fistula	2.6	1.3	0.39 *
Atrioventricular block III°	0.3	0.4	1.00 *
Pericardial effusion	1.0	1.7	0.49 *
**Minor bleeding**	6.7	2.1	0.008 *
**Medication at discharge**			
**Betablocker**	79.0	73.2	0.095
**Digitalis**	4.7	5.4	0.66
**AAD**	60.1	48.1	<0.01
Class I	26.8	30.1	0.36
Class III	33.9	18.0	<0.001
**Statin**	39.4	13.8	<0.001
**Diuretics**	31.3	14.2	<0.001
**ACE-inhibitors/ARB**	62.2	27.6	<0.001
**Clopidogrel**	2.8	0.4	0.031
**Aspirin**	10.9	8.4	0.31
**Vitamin K antagonists**	92.0	91.2	0.74

AAD = antiarrhythmic drug; ACE = angiotensin-converting-enzyme; ARB = angiotensin-II-receptor-blockers; Data are presented as percentages of patients. * *p*-values calculated by Fisher’s exact test.

**Table 3:** Medication at long-term follow-up interview.

	HTN (*n* = 354) (%)	No HTN (*n* = 223) (%)	*p* Value
**Antiarrhythmic medication**	33.4	28.5	0.22
Class I	12.8	13.6	0.80
Class III	19.8	15.4	0.19
**Beta-blocker**	75.6	60.3	<0.001
**Digitalis**	2.8	4.7	0.28
**Diuretics**	33.1	17.8	<0.001
**ACE-inhibitors/ARBs**	65.7	30.8	<0.001
**Statins**	32.0	12.6	<0.001
**Oral anticoagulation**	45.1	33.6	0.008

ACE = angiotensin converting enzyme; ARB = angiotensin II receptor blocker; Data are presented as percentages of patients.

**Table 4:** Differences in outcomes at follow-up according to AF type.

	HTN (%)	No HTN (%)	Adjusted Odds Ratio (95%–CI) *	*p* Value *	*p* Value for Interaction
**Rehospitalization rates**					
Paroxysmal AF (*n* = 225/155)	43.6	40.0	0.98 (0.63–1.53)	0.94	0.080
Persistent AF/Long-standing persistent AF (*n* = 121/58)	58.7	37.9	1.97 (1.02–3.81)	0.045	
**Cardiovascular rehospitalization**					
Paroxysmal AF (*n* = 225/155)	33.8	30.3	1.00 (0.62–1.59)	0.99	0.172
Persistent AF/Long-standing persistent AF (*n* = 121/58)	49.6	32.8	1.74 (0.89–3.42)	0.106	
**NYHA II+**					
Paroxysmal AF (*n* = 220/143)	29.5	20.3	1.11 (0.65–1.91)	0.70	0.152
Persistent AF/Long-standing persistent AF (*n* = 120/58)	45.0	24.1	2.15 (1.03–4.51)	0.042	
**Angina pectoris**					
Paroxysmal AF (*n* = 222/151)	16.2	11.3	1.23 (0.63–2.39)	0.54	0.051
Persistent AF/Long-standing persistent AF (*n* = 121/55)	18.2	1.8	10.32 (1.33–79.97)	0.025	

NYHA = New York Heart Association. Data are presented as percentages of patients. * adjusted for age, sex, coronary artery disease, diabetes, renal insufficiency and peripheral artery disease.
